# Prediction Score for Cervical Spine Fracture in Patients with Traumatic Neck Injury

**DOI:** 10.1155/2021/6658679

**Published:** 2021-03-18

**Authors:** Natsinee Athinartrattanapong, Chaiyaporn Yuksen, Sittichok Leela-amornsin, Chetsadakon Jenpanitpong, Sirote Wongwaisayawan, Pittavat Leelapattana

**Affiliations:** ^1^Department of Emergency Medicine, Faculty of Medicine Ramathibodi Hospital, Mahidol University, Bangkok, Thailand; ^2^Department of Radiology, Faculty of Medicine Ramathibodi Hospital, Mahidol University, Bangkok, Thailand; ^3^Department of Orthopedics, Faculty of Medicine Ramathibodi Hospital, Mahidol University, Bangkok, Thailand

## Abstract

**Background:**

Cervical spine fracture is approximately 2%–5%. Diagnostic imaging in developing countries has several limitations. A computed tomography scan is not available 24 hours and not cost-effective. This study aims to develop a clinical tool to identify patients who must undergo a computed tomography scan to evaluate cervical spine fracture in a noncomputed tomography scan available hospital.

**Methods:**

The study was a diagnostic prediction rule. A retrospective cross-sectional study was conducted between August 1, 2016, and December 31, 2018, at the emergency department. This study included all patients aged over 16 years who had suspected cervical spine injury and underwent a computed tomography scan at the emergency department. The predictive model and prediction scores were developed via multivariable logistic regression analysis.

**Results:**

375 patients met the criteria. 29 (7.73%) presented with cervical spine fracture on computed tomography scan and 346 did not. Five independent factors (i.e., high-risk mechanism of injury, paraparesis, paresthesia, limited range of motion of the neck, and associated chest or facial injury) were considered good predictors of C-spine fracture. The clinical prediction score for C-spine fracture was developed by dividing the patients into three probability groups (low, 0; moderate, 1–5; and high, 6–11), and the accuracy was 82.52%. In patients with a score of 1–5, the positive likelihood ratio for C-spine fracture was 1.46. Meanwhile, those with a score of 6–11 had an LR+ of 7.16.

**Conclusion:**

In a noncomputed tomography scan available hospital, traumatic spine injuries patients with a clinical prediction score ≥1 were associated with cervical spine fracture and should undergo computed tomography scan to evaluate C-spine fracture.

## 1. Introduction

Traumatic spine injury (TSI) may cause cervical spine (C-spine) fracture, subluxation, dislocation, or cervical cord injury due to various mechanisms [1]. The Advanced Trauma Life Support (ATLS)® recommends the use of the National Emergency X-Radiography Utilization Study (NEXUS) [2] criteria and the Canadian C-spine Rule (CCR) [3], which are two clinical decision tools used by clinicians in identifying patients with a lower risk for clinically important C-spine injury. Therefore, the use of imaging modalities and cervical motion restriction equipment will no longer be required [1, 4, 5]. The NEXUS criteria' sensitivity and specificity are 0.83–1.00 and 0.02–0.46, respectively, and the positive and negative predictive values are 1.44 and 0.3, respectively. Meanwhile, the sensitivity and specificity of CCR are 0.90–1.00 and 0.01–0.77, respectively, and the positive and negative predictive values are 1.69 and 0.18, respectively [6–8].

Computed tomography (CT) scan improves the accuracy of diagnosing C-spine fracture. However, it has several limitations, including its high cost, unavailability of equipment, and radiologists interpreting the results. Thus, this imaging modality can be performed only in secondary and tertiary care centers. In C-spine injury cases, additional assessments should be based on the appropriateness criteria [9], and patients suspected of C-spine fracture should be assessed appropriately.

In developing countries or non-CT scan available hospitals, plain film radiography of the C-spine was used to diagnose the C-spine injury. However, it is not useful in the detection of C-spine fracture [10]. This study aims to develop a clinical prediction score for C-spine fracture in patients with TSI for non-CT scan available hospitals to select the TSI patients sent to CT scan.

## 2. Methods

This was a diagnostic prediction rule. A retrospective cross-sectional study was conducted between August 1, 2016, and December 31, 2018, at the emergency department (ED) of Ramathibodi Hospital, a university-affiliated super tertiary care hospital in Bangkok, Thailand. The patients who visited the ED were about 3,500 patients per month, 10% was traumatic patient, and about 20 patients per month were sent to the cervical spine CT scan.

The adult patients (aged ≥16 years) with C-spine injury (traumatic neck injury) who underwent CT scans at the ED were included in the study. The patients were categorized into two groups based on their C-spine CT scan results. The first group had positive C-spine CT scan results (C-spine fracture, subluxation, dislocation, and traumatic spondylolisthesis). Meanwhile, the second group had negative C-spine CT scan results. A radiologist officially assessed all emergency C-spine CT scan results.

### 2.1. Sample Size

On the basis of a previous pilot study, the incidence rate of C-spine fracture in the ED was 5.88% (1 : 17). In our study, the sample size was calculated via a one-sample comparison of proportions, with an alpha of 0.05 (one side), power of 0.8, and *n*2/*n*1 of 17.

### 2.2. Statistical Analysis

The potential predictors were compared to identify differences (*p* value) in clinical characteristics using the *t*-test and exact probability test. The predictive factors were individually calculated via a univariable logistic regression analysis and were presented as an area under the receiver operating characteristic (AUROC) curve and 95% confidence interval (95% CI). The clinical predictors that had a high discriminative performance (AUROC curve), *p* value, and clinical relevance were divided into two categories by calculating odds ratio (OR) via a multivariable logistic regression analysis.

The calibration of the prediction was presented using the Hosmer–Lemeshow test. The score was used to predict the risk of C-spine fracture, and the observed risks were presented in the graph. The number and percentage of each group, positive likelihood ratio (LR+), 95% CI, and *p* value were observed.

### 2.3. Ethics Statement

This study was approved by the committee on Human Rights Related to Research Involving Human Subjects, Thailand (ID 05-61-68).

## 3. Results

A total of 375 patients with suspected C-spine injury underwent CT scan. The demographic characteristics at baseline between the groups with and without C-spine fractures are demonstrated in Table 1. The study variables might be the clinical prognostic factors of C-spine fracture. A significant difference was observed in terms of falling in the ground (13.79% vs. 34.9%, *p* value = 0.023), fall from a height (20.69% vs. 6.16%, *p* value = 0.013), high-speed collision (6.9% vs. 0.29%, *p* value = 0.017), high-risk mechanism (37.93% vs. 14.2%, *p* value = 0.002), paraparesis (37.04% vs. 11.28%, *p* value = 0.001), paresthesia (19.23% vs. 4.38%, *p* value = 0.009), cannot ambulate in the ED (37.93% vs. 15.07%, *p* value = 0.004), and limited ROM of the neck (31.03% vs. 9.86%, *p* value = 0.003).

The clinical prognostic factors that had a high discriminative performance (AUROC curve) were high-risk mechanism, associated chest or facial injury, paraparesis, paresthesia, and limited ROM of the neck.

The basis of the multivariable analysis between the groups with and without C-spine fractures is given in Table 2. The significant prognostic factors of C-spine fracture were associated with chest or facial injury (OR: 2.72, 95% CI: 1.11–6.66, *p* value = 0.029), high-risk mechanism (OR: 2.94, 95% CI: 1.1–7.87, *p* value = 0.031), paraparesis (OR: 5.38, 95% CI: 1.33–21.7, *p* value = 0.018), paresthesia (OR: 1.66 95% CI: 0.3–9.22, *p* value = 0.565), and limited ROM of the neck (OR: 3.77 95% CI: 1.39–10.18, *p* value = 0.009), and the item score ranged from 0 to 3.

**Figure 1:**
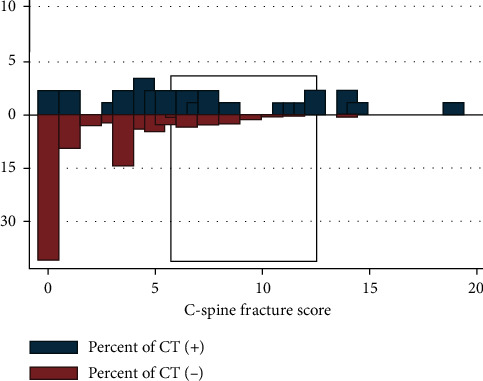
Two-way bar graph of the C-spine fracture score: CT (+) (*n* = 29) versus CT (−) (*n* = 346).

**Figure 2:**
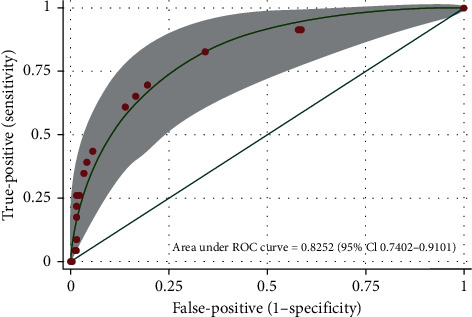
Area under the receiver operating characteristic (AUROC) curve of the clinical risk score and 95% confidence interval (95% CI) for the prediction of cervical spine fracture.

The strength of the clinical prediction score in identifying positive results (C-spine fracture) based on the CT scan results was presented as the distributional plot of the clinical risk score (Figure 1) and the AUROC curve (82.52% (95% CI: 74.02–91.01)) (Figure 2).

**Figure 3:**
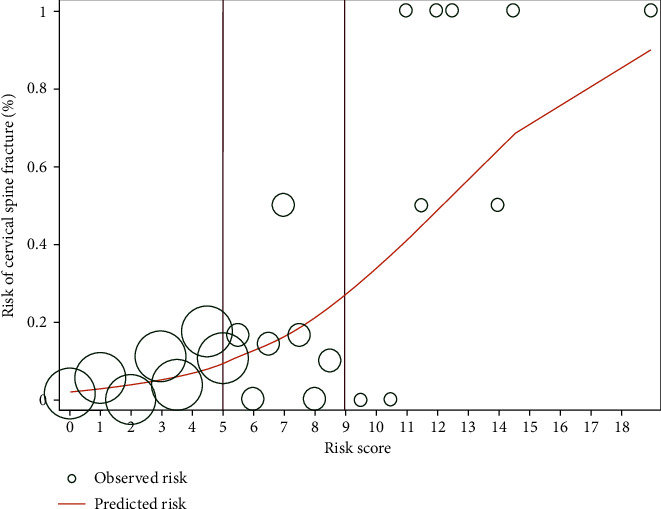
Observed risk (circle) versus score-predicted risk (solid line) of cervical spine fracture.

Figure 3 shows the observed risk (circle) and the score-predicted risk (solid line) of C-spine fracture. The score-predicted risk of C-spine fracture increased closely proportional to that of the observed risk.

The clinical prediction scores for C-spine fracture are given in Table 3, divided into three categories: scores of 0, low probability; scores of 1–5, moderate probability; and scores of 6–11, high probability. The LRs for a positive C-spine fracture on CT scan was 0.18 (95% CI: 0.05–0.56) in the low probability category, 1.46 (95% CI: 1.09–1.96) in the moderate probability category, and 7.16 (95% CI: 2.82–18.19) in the high probability category.

## 4. Discussion

Our study was conducted in a super tertiary care facility, and each patient with traumatic neck injury underwent emergency C-spine CT scan. Only 7.73% of the patients presented with C-spine fracture on CT scan. The significant clinical predictors for C-spine fracture were the NEXUS [3] (paresthesia and paraparesis) and CCR [4] (high-risk mechanism, paresthesia, and limited ROM of the neck). Associated chest and facial injuries were considered significant clinical predictors, and this result was in accordance with that of the study of Clayton JL [2]. Each patient with C-spine fracture had an associated head injury. However, it was not significantly associated with C-spine fracture in our study (*p* value = 0.389).

In the univariable analysis, the significant predictors were identified on the basis of significance (*p* value) and high discriminative performance (AUROC curve). In the multivariable analysis, the best clinical predictors were associated with chest or facial injury, high-risk mechanism, paraparesis, paresthesia, and limited ROM of the neck.

In our study, patients with C-spine injury (traumatic neck injury) who had a score of 0 were classified under the low probability category with an LR + of 0.18, which indicates a low risk of C-spine fracture. In this category, patients in a non-CT scan available hospital will not require referral for further imaging except if no other obvious clinical signs of an injury were observed.

Patients with C-spine injury who had a score of 1–5 were classified under the moderate probability category with an LR+ of 1.46, which indicates a moderate risk of C-spine fracture. In this category, patients in a non-CT scan available hospital should be referred to tertiary care centers for the evaluation of C-spine fracture. The use of plain film radiography of the neck in three views (PA, lateral, and open-mount) as an adjunct was not effective in assessing C-spine fracture [11].

Last, patients with a score of 6–11 were classified under the high probability category with an LR+ of 7.16, which indicates a high risk of C-spine fracture. Thus, patients under this category must be immediately transferred to any hospital with an available CT scan to facilitate the evaluation of C-spine fracture and to prevent cervical motion using motion restriction equipment.

We implement the score for selecting the TSI patients to CT scan in Ramathibodi Hospital and promote this score in other hospitals. We plan to do the other research studies for external validation of the score. We are using the score to select the cervical spine injuries patients in Ramathibodi Hospital sent to CT scan. C-spine CT scans in patients with moderate and high risks were associated with decrease costs of $26,800–$2,250 per year.

According to our score, the patient's score of 0, without the cervical injury hypothesis, should not require a CT scan in the exclusive occasions, if no predictors' variables were present. According to other studies, patients with paresthesia after neck injury are at moderate to high risks for C-spine fractures, subluxation, dislocation, or traumatic spondylolisthesis [12, 13]. We include the variable of paresthesia (1 point) in the moderate risk group, and they require a CT scan.

There are some limitations to this study. The efficacy of our clinical prediction score when used in tertiary care facilities was assessed via a retrospective chart review. Thus, some background data may not be complete. All patients with C-spine injury in our hospital underwent CT scan based on the protocol in recent years. Thus, we cannot collect previous data before the protocol was launched. The total number of patients who were enrolled in this study was lower than the calculated sample size. However, results showed that even though a small number of cases were included, the significant factors for the prediction of C-spine fracture were identified. We suggest further validation studies of our score to use in other settings.

In conclusion, a clinical prediction score ≥1 was associated with C-spine fracture. Patients under the moderate and high probability categories in a non-CT scan available hospital should be sent to CT scan for the evaluation of C-spine fracture.

## Figures and Tables

**Table 1:** Clinical prognostic factors of CT (+) versus CT (−), *p* value, AUROC, and 95% CI.

Prognostic factors	CT (+), *n* = 29		CT (−), *n* = 346		*p* value	AUROC (95% CI)	
	*n*	%	*n*	%			
Gender							
Male	13	44.8	212	61.3	0.113	0.582	0.487–0.678

Age (years)							
≥65	11	37.9	124	35.8	0.842	0.511	0.417–0.604

Mechanism of injury							
Motor vehicle collision	7	24.1	87	25.5	1.000	0.493	0.410–0.576
Pedestrian injury	2	6.9	16	4.7	0.642	0.511	0.463–0.559
Assault/violence	0	0	29	8.6	0.149	0.457	0.442–0.472
Falling to the ground	4	13.8	119	34.9	0.023	0.395	0.326–0.463
Fall from a height	6	20.7	21	6.2	0.013	0.573	0.497–0.949

Associated injury							
Head	29	100	312	94.0	0.389	0.530	0.517–0.543
Facial	10	34.5	61	18.4	0.049	0.581	0.490–0.671
Chest	8	27.6	43	13.0	0.046	0.573	0.488–0.658
Abdomen	2	6.9	10	3.0	0.249	0.519	0.472–0.576
Pelvic injury	2	6.9	9	2.7	0.218	0.521	0.473–0.569
Other spinal/vertebral injuries	3	10.3	11	3.3	0.093	0.535	0.478–0.592

History							
Intoxication	5	17.2	54	16.4	1.000	0.504	0.431–0.577
Pain in the posterior neck	7	24.1	101	30.7	0.532	0.467	0.384–0.550
Painful distracting injury	2	6.9	30	9.1	1.000	0.489	0.439–0.538
High-risk mechanism	11	37.9	49	14.2	0.002	0.619	0.527–0.710
Immediate onset of neck pain	6	20.7	76	22.0	1.000	0.493	0.415–0.571
Unable to rotate the neck normally	18	62.1	117	34.6	0.005	0.637	0.544–0.731

Physical examination							
GCS score: 15	17	58.6	251	73.0	0.218	0.570	0.475–0.664
GCS score: 13-14	6	20.7	47	13.7			
GCS score: 9–12	5	17.2	30	8.7			
GCS score: ≤8	1	3.5	16	4.7			

Paraparesis	10	37.0	38	11.3	0.001	0.629	0.534–0.723
Paresthesia	5	19.2	14	4.4	0.009	0.574	0.496–0.652
Cannot sit in the ED	12	41.4	91	26.4	0.088	0.575	0.481–0.669
Cannot ambulate	11	37.9	52	15.1	0.004	0.614	0.522–0.706
Midline C-spine tenderness	10	34.5	143	41.5	0.557	0.465	0.373–0.557
Limited neck range of motion	9	31.0	34	9.9	0.003	0.606	0.519–0.693

AUROC, area under the receiver operating characteristic; 95% CI, 95% confidence interval; CT, computed tomography; GCS, Glasgow Coma Scale; ED, emergency department.

**Table 2:** Significant predictors of cervical spine fracture and assigned item score.

Predictors	Category	OR	95% CI	*p* value	Coefficient^*∗*^	Score
Associated chest or facial	No	1.00	Reference	—	—	0
	Yes	2.72	1.11–6.66	0.029	1.00	2

High-risk mechanism	No	1.00	Reference	—	—	0
	Yes	2.94	1.10–7.87	0.031	1.08	2

Paraparesis	No	1.00	Reference	—	—	0
	Yes	5.38	1.33–21.70	0.018	1.68	3

Paresthesia	No	1.00	Reference	—	—	0
	Yes	1.66	0.30–9.22	0.565	0.50	1

Limited neck ROM	No	1.00	Reference	—	—	0
	Yes	3.77	1.39–10.18	0.009	1.33	3

^*∗*^Coefficients based on the multivariable continuation ratio logistic regression. OR, odds ratio; 95% CI, 95% confidence interval; ROM, range of motion.

**Table 3:** Distribution of CT (+) versus CT (−) according to low, moderate, and high probability categories, LR+, and 95% CI.

Probability categories	Scores	CT (+), *n* = 27		CT (−), *n* = 322		LR+	95% CI	*p* value
		*n*	%	*n*	%			
Low	0	1	3.7	165	51.2	0.18	0.05–0.56	<0.001
Moderate	1–5	20	74.1	147	45.7	1.46	1.09–1.96	0.044
High	6–11	6	22.2	10	3.1	7.16	2.82–18.19	<0.001
Mean ± SD		1.51 ± 1.85		3.81 ± 2.47				<0.001

LR+, positive likelihood ratio; 95% CI, 95% confidence interval; SD, standard deviation; CT, computed tomography.

## Data Availability

The data used to support the findings of this study are available from the corresponding author upon request.

## Additional Points

Cervical spine fracture diagnostic by computed tomography scan in developing countries has limitations. Canadian C-Spine Rule (CCR) used in a developing country has limitations. There is no clinical prediction tool to identify patients who must undergo a CT scan in developing countries. In a noncomputed tomography scan available hospital, patients should be sent to CT scan for the evaluation of C-spine fracture. Traumatic spine injuries in patients with a clinical prediction score ≥1 were associated with cervical spine fracture and should undergo computed tomography scan to evaluate C-spine fracture.
